# Erratum: Sustained Post-Developmental T-Bet Expression Is Critical for the Maintenance of Type One Innate Lymphoid Cells *In Vivo*

**DOI:** 10.3389/fimmu.2021.822468

**Published:** 2021-12-10

**Authors:** Frontiers Production Office

**Affiliations:** Frontiers Media SA, Lausanne, Switzerland

**Keywords:** T-bet, innate lymphoid cells, ILCs, intestinal inflammation, mucosal homeostasis 4

Due to a production error, Emily Read was incorrectly affiliated to “Department of Diabetes, School of Life Course Sciences, Faculty of Life Sciences and Medicine, King’s College, London, United Kingdom” in the online version of the article.

The publisher apologizes for this mistake. The original version of this article has been updated.

