# Supplementary figures and images for: Sustained Post-Developmental T-Bet Expression Is Critical for the Maintenance of Type One Innate Lymphoid Cells In Vivo
Source: Front Immunol. 2021 Oct 29;12:760198. doi: 10.3389/fimmu.2021.760198 (PMC8594445; doi:10.3389/fimmu.2021.760198)

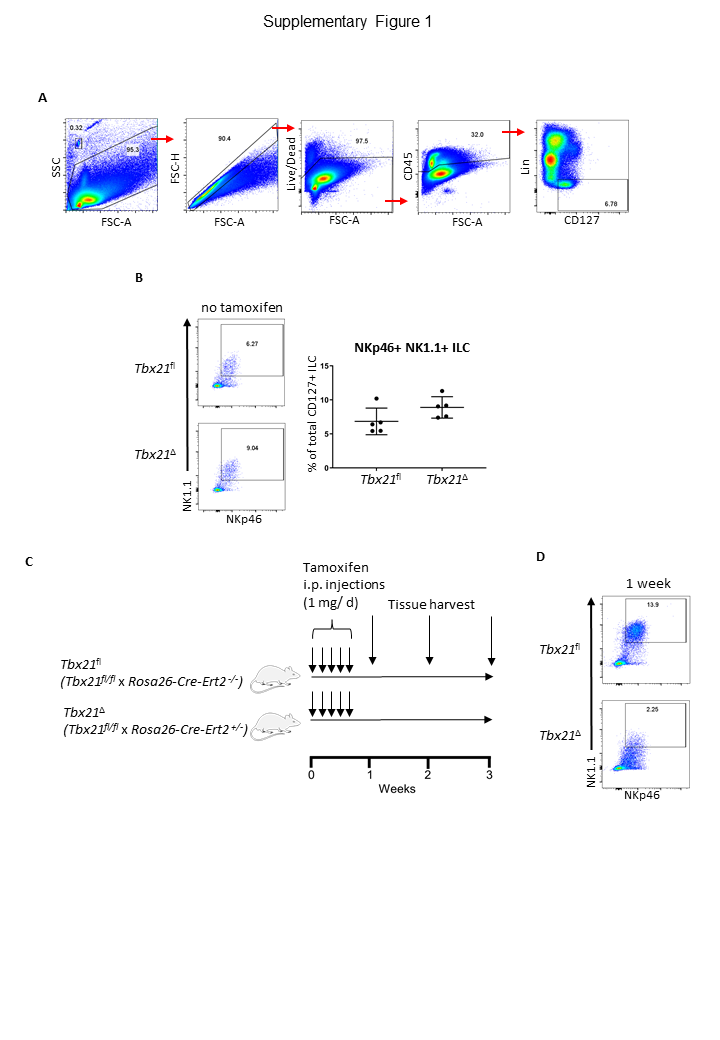

Supplement: Supplementary Figure 1 — Analysis of cLP ILC1 depletion at days 0 and 7 post onset of induced T-bet depletion. cLP ILC were isolated from untreated or tamoxifen-treated Tbx21fl and Tbx21 Δ mice for flow cytometry analysis. (A) ILC were gated as live CD45+ Lin- CD127+ leukocytes. (B, D) NKp46+ NK1.1+ ILC (B) from untreated mice (n=5) or (D) 7 days after the first injection of tamoxifen (n=1). (C) Model of induced depletion of T-bet using Tbx21 Δ and Tbx21fl control mice. [file Image_1.tif]

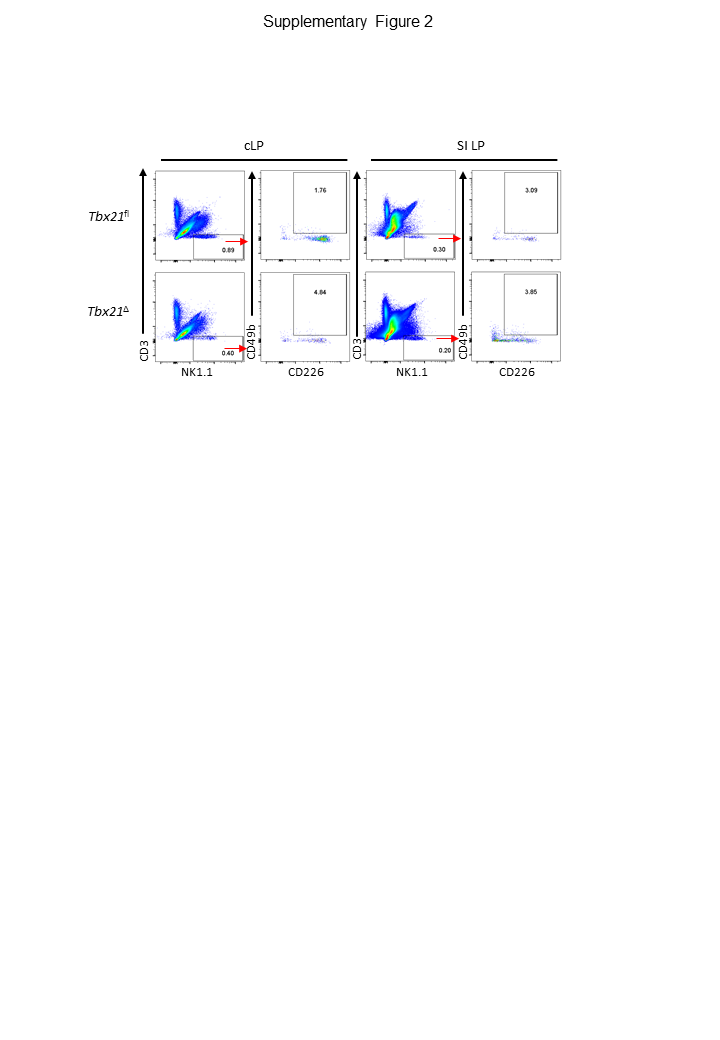

Supplement: Supplementary Figure 2 — Induced T-bet depletion in vivo does not affect intestinal CD49b+ NK cells. NK cells were isolated from tamoxifen-treated Tbx21fl and Tbx21 Δ mice for flow cytometry analysis 21 days after the first injection of tamoxifen. cLP and SI LP NK cells were analysed as live CD45+ CD3- NK1.1+ CD226+ CD49b+ leukocytes (n=1). [file Image_2.tif]

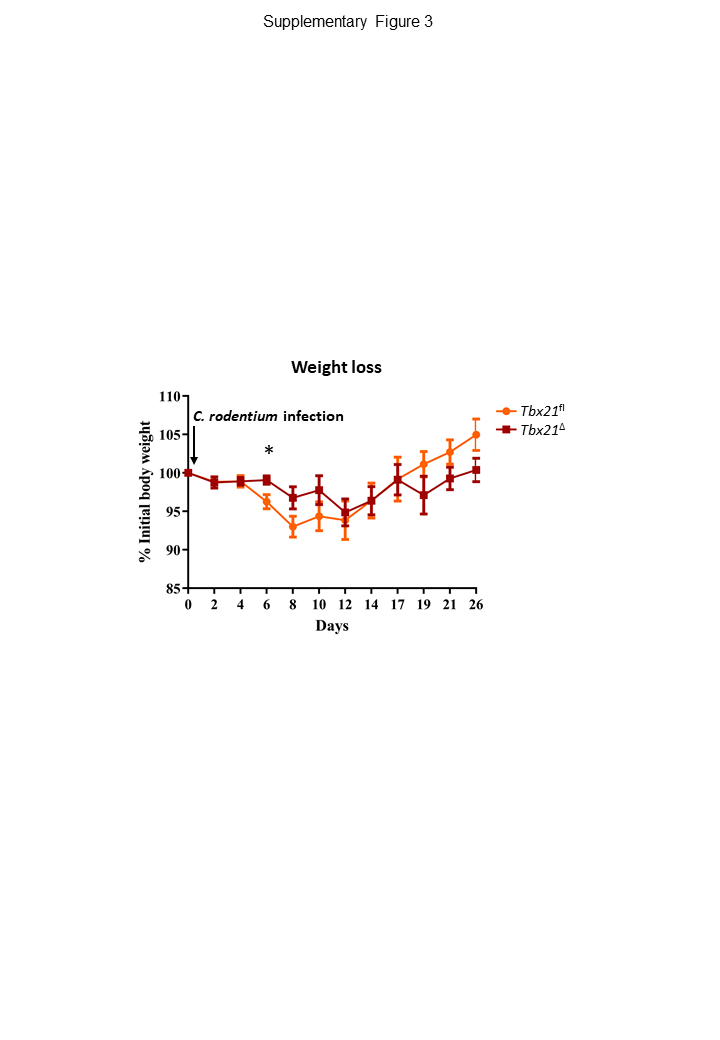

Supplement: Supplementary Figure 3 — Induced T-bet depletion has limited effect on colitis caused by C. rodentium-infection. Tamoxifen pre-treated Tbx21fl and Tbx21 Δ mice were infected with C. rodentium 3 weeks after the first injection of tamoxifen for infection analysis. Animal weight difference in relation to day 0 on days post infection are shown. Data shown are representative of 8-9 biological replicates. [file Image_3.tif]

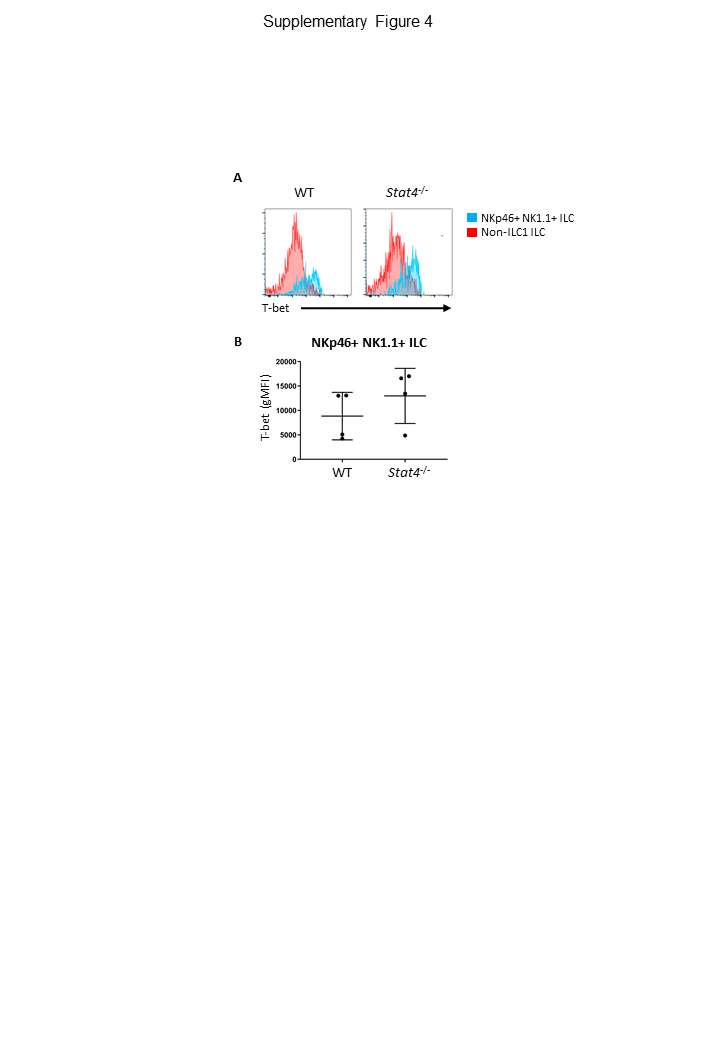

Supplement: Supplementary Figure 4 — T-bet-expression in STAT4-deficient ILC. cLP leukocytes were isolated from WT or Stat4-/- mice. (A) Flow cytometry analysis of T-bet in cLP NKp46+ NK1.1+ ILC and non-ILC1 cLP ILC and statistical analysis of T-bet expression in cLP NKp46+ NK1.1+ ILC are illustrated. [file Image_4.tif]

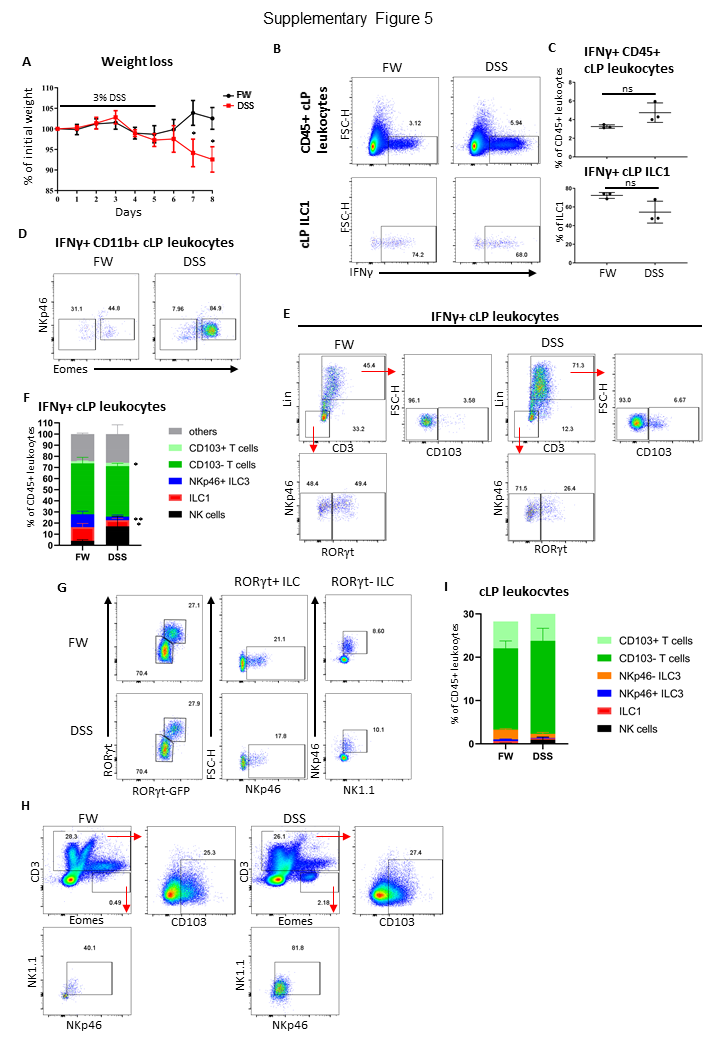

Supplement: Supplementary Figure 5 — T-bet-expressing ILC are a relevant source of IFNγ at steady state. Rorc GFP mice received 3% DSS for 5 days followed by a period of rest. (A) Daily percentual weight change to body weight at the start are illustrated. (B–H) cLP leukocytes were isolated 3 days after DSS withdrawal and re-stimulated with PMA and ionomycin for 3 hours prior to analysis. (B) Flow cytometry analysis of IFNγ expressing live CD45+ leukocytes and RORγt- NKp46+ NK1.1+ ILC1 and (C) respective statistical analyses are shown. Frequency of (D) CD11b+ Eomes+ NKp46+ NK, (E) CD11b- CD103- CD3+ T cells, CD11b- CD103+ CD3+ T cells, RORγt- NKp46+ ILC1 and RORγt+ NKp46+ ILC3 among IFNγ+ CD45+ leukocytes from DSS-treated and control mice and (F) respective statistical analysis are illustrated. (G) Flow cytometry analysis of RORγt- NKp46+ NK1.1+ ILC and RORγt+ NKp46+ ILC3, (H) Eomes+ NKp46+ NK and CD103+ and CD103- T cells and (I) a summary plot of CD45+ leukocyte subset frequencies are shown. Data shown are representative of 3 biological replicates. [file Image_5.tif]

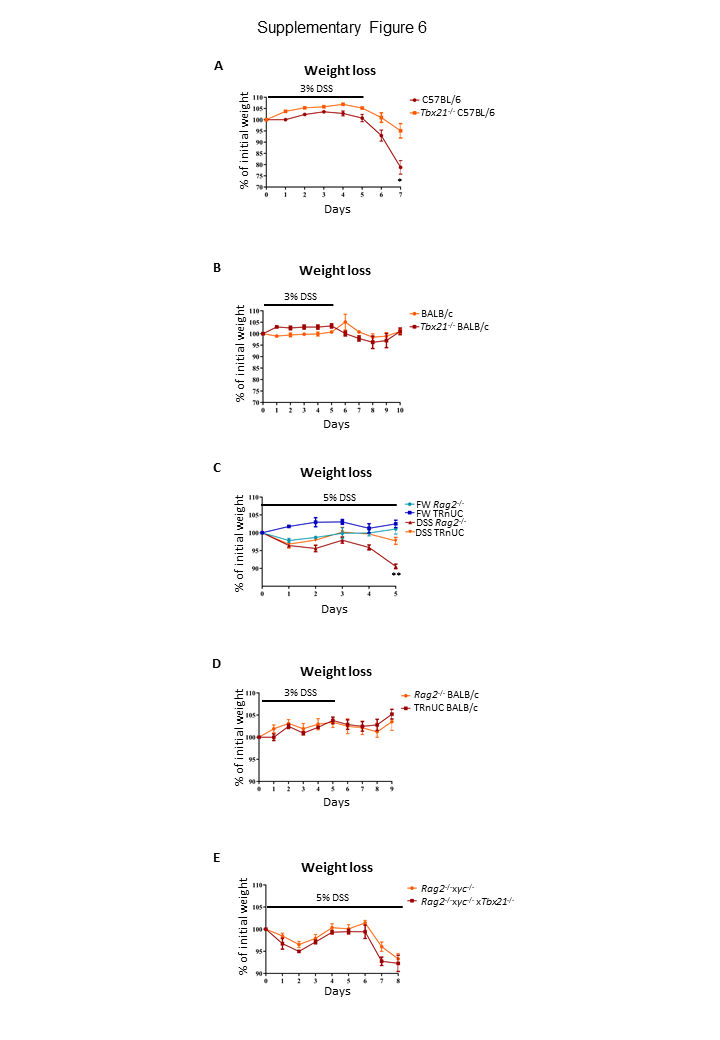

Supplement: Supplementary Figure 6 — Tbx21-/- mice develop milder DSS-elicited colitis. Mice received DSS in the drinking water for weight loss analysis. (A) C57BL/6 and Tbx21-/- C57BL/6 mice or (B) BALB/c and Tbx21 -/- BALB/c mice received 3% DSS for 5 days followed by a period of rest (n=4). Rag2 -/- and TRnUC received (C) 5% DSS and fresh water (FW) for 5 days (n=3) or (D) 3% DSS for 5 days followed by a period of rest (n=4). (E) Rag -/-xγc -/- and Rag -/-xγc -/-xTbx21 -/- mice received 5% DSS for 8 days (n=4). Daily recording of weight change in comparison to the starting point are illustrated. [file Image_6.tif]

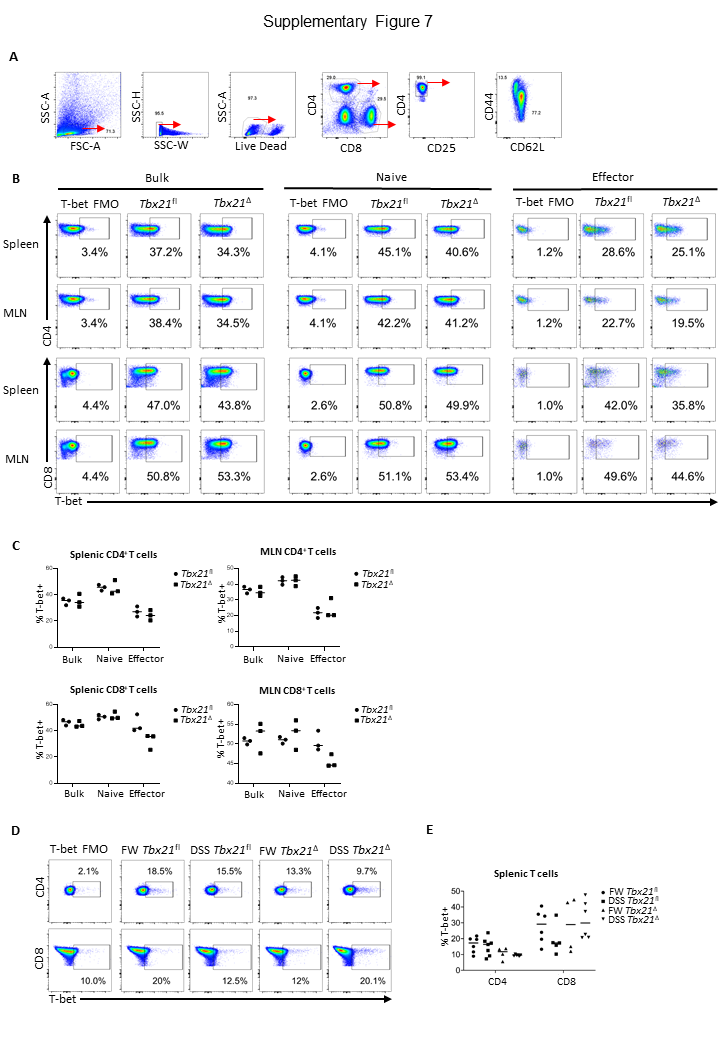

Supplement: Supplementary Figure 7 — Induced T-bet depletion in vivo limits, but does not restrict the potency to generate Th1 cells in vivo. Leukocytes from MLN and spleen of tamoxifen-treated Tbx21fl and Tbx21 Δmice were isolated for flow cytometry analysis 3 weeks after the first injection. (A) Gating strategy for naïve (CD44hi CD62L-) and effector (CD44low CD62L+) CD4+ and CD8+ T cells. (B) Expression of T-bet and (C) statistical analysis of its expression in MLN and splenic bulk, naïve and effector CD4+ and CD8+ T cells are shown (n=3). (D, E) Tamoxifen-pretreated Tbx21fl and Tbx21 Δmice were exposed to 3% DSS for 5 days and splenic leukocytes were isolated another 5 days later for flow cytometry analysis of CD3+ T cells. (D) T-bet percentage frequency in CD4+ and CD8+ T cells and (E) statistical analysis of T-bet expression in these cells are shown (n=4-6). [file Image_7.tif]

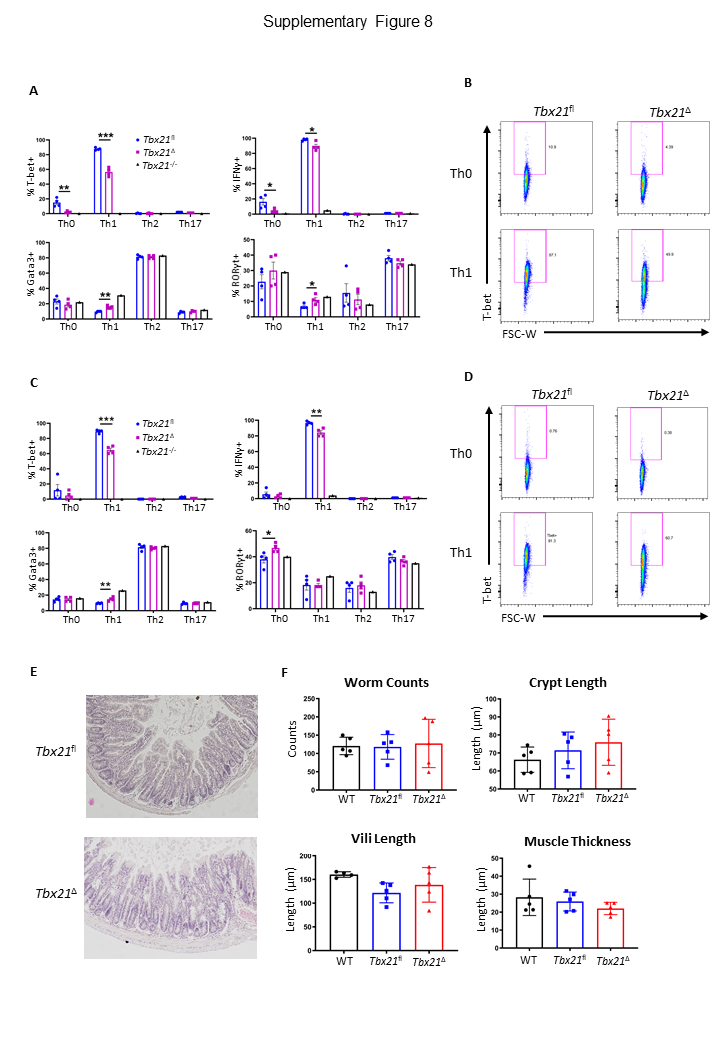

Supplement: Supplementary Figure 8 — Induced T-bet depletion in vivo limits, but does not restrict the potency to generate Th1 cells in vitro and does not alter the immune response to T. spiralis. T cells were isolated from the spleen of tamoxifen-treated Tbx21fl and Tbx21 Δ mice 3 weeks after the first injection for polarization assays and flow cytometry analysis. (A) T-bet, IFNγ, GATA3 and RORγt expression in (A, B) MLN and (C, D) splenic CD4+ T cells upon Th0, Th1, Th2 and Th17 polarization is demonstrated. (A, C) Percentage of marker expression and (B, D) T-bet expression are shown (n=4). Tamoxifen pre-treated Tbx21fl and Tbx21 Δ mice were infected 3 weeks after the first injection with T. spiralis for infection analysis. (E) Histology, (F) worm burden, crypt length, vili length and muscle thickness are shown (n=5). [file Image_8.tif]

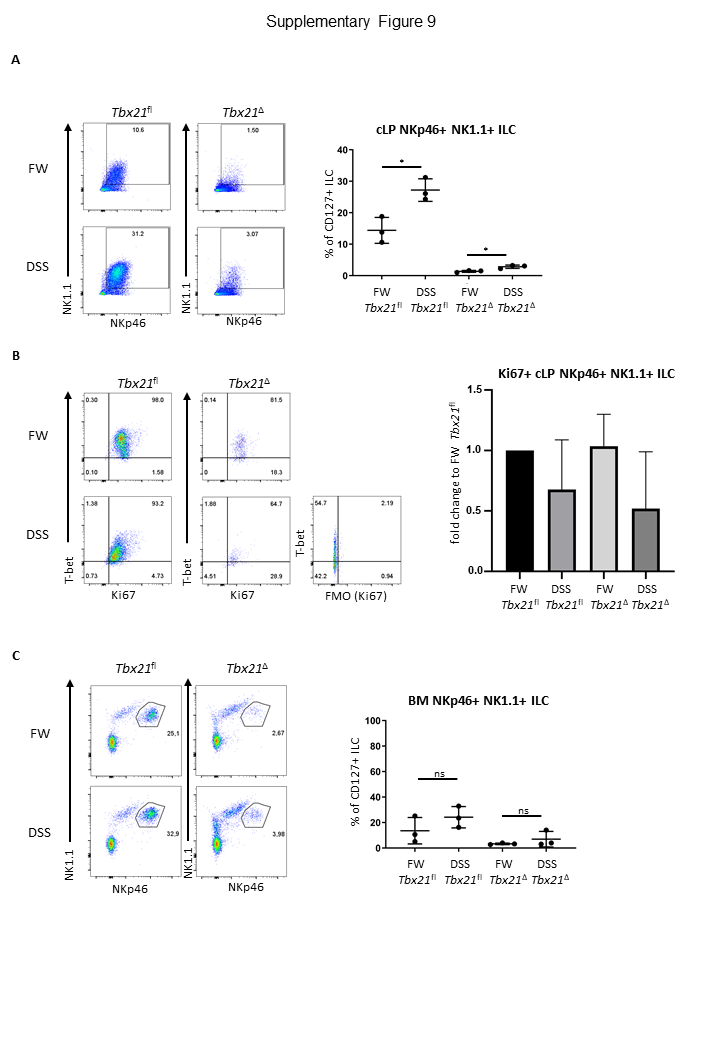

Supplement: Supplementary Figure 9 — Bone marrow-derived NKp46+ ILC can recolonize the intestine upon induced depletion of cLP ILC1. DSS colitis was induced in Tbx21fl and Tbx21 Δ mice pre-treated with tamoxifen (i.e. 21 days after the first injection). Mice were exposed to 3% DSS for 5 days and another 3 days later tissues were isolated and CD127+ NKp46+ NK1.1+ ILC were analysed by flow cytometry. In control settings mice received fresh water without DSS. (A) Flow cytometry analysis of cLP NKp46+ NK1.1+ ILC percentage share of CD127+ ILC. (B) Ki67 and T-bet expression in cLP NKp46+ NK1.1+ ILC and fold change difference of Ki67 expression in NKp46+ NK1.1+ ILC relating to fresh water-exposed Tbx21fl mice are illustrated. (C) Cellularity of live CD45+ Lin- CD127+ NKp46+ NK1.1+ ILC in the bone marrow at day 3 of DSS withdrawal and the frequency of BM NKp46+ NK1.1+ ILC within total BM CD127+ ILC are demonstrated. Data shown are representative of 3 biological replicates. [file Image_9.tif]
